# Effect of acupuncture on asthma control and body weight changes in obese patients with asthma

**DOI:** 10.3389/fmed.2026.1861336

**Published:** 2026-07-03

**Authors:** Zhihong Shi, Qing Quan, Xiaohong Bai, Yanan Wang, Xiao Lai, Xiaoyan Hu

**Affiliations:** 1 Mongolian Medical Department, Affiliated Hospital of Inner Mongolia Medical University, Hohhot, China; 2 General Surgery, Xing'an League Mongolian Medicine Hospital, Ulanhot City, China; 3 College of Mongolian Medicine, Inner Mongolia Minzu University, Tongliao, China; 4 Imaging Department, Xilingol League Mongolian Medical Hospital, Xilinhot, China; 5 Brucellosis, International Mongolian Hospital, Hohhot, China; 6 Internal Medicine, Hangjin Banner Mongolian Medical General Hospital, Ordos, China

**Keywords:** acupuncture, asthma, asthma control, body weight, obesity

## Abstract

**Background:**

Chronic persistent asthma is difficult to control and is often comorbid with obesity. Acupuncture has been shown to reduce airway inflammation and improve lung function, and it may also help regulate body weight to some extent. Currently, obesity combined with chronic persistent asthma is a research hotspot, but interventional studies involving acupuncture remain relatively limited. This study aims to fill this gap.

**Methods:**

Patients were divided into a control group (conventional western medical treatment) and a treatment group (conventional western medical treatment plus acupuncture). Baseline characteristics between the two groups were compared using univariate analysis. Changes before and after treatment were assessed for pulmonary function indicators (PEF, FEV1, FEV1/FVC), Asthma Control Test (ACT) scores, Asthma Quality of Life Questionnaire (AQLQ) scores, serum inflammatory markers (IL-17, CRP), and BMI. Multivariate logistic regression and multiple linear regression analyses were conducted to evaluate the effects of treatment modality on asthma control and body weight changes. A response model was constructed to predict asthma control outcomes, and its performance was evaluated using the ROC curve. Further stratified analyses were performed to assess the predictive ability of the model in different subgroups.

**Results:**

After treatment, the treatment group showed improvements in pulmonary function, asthma control, quality of life, inflammatory markers, and BMI. Treatment modality was independently associated with asthma control and weight changes. Asthma severity, comorbidities, baseline FEV1, and baseline CRP levels were also associated with asthma control. The response model demonstrated moderate discriminative ability (AUC = 0.656), with stronger effects observed in patients aged ≥50 years, males, and those without acute exacerbations in the past year.

**Conclusion:**

This study provides a new intervention strategy and supporting evidence for individualized treatment in obese patients with chronic persistent asthma.

## 1 Introduction

Bronchial asthma is a common chronic inflammatory disease affecting the airways, clinically characterized by recurrent episodes of dyspnea, wheezing, and airway obstruction (1, 2). It is estimated that approximately 260 million people worldwide are affected by asthma (3). Epidemiological studies in China indicate that among adults aged 20 years and above, the prevalence of asthma with wheezing is about 4.2%, corresponding to approximately 45.7 million adult asthma patients (4). This highlights that asthma remains a major global public health issue. The age group of 0–14 years is one of the peak periods of incidence, and childhood asthma is often associated with allergic diseases such as eczema and allergic rhinitis (5, 6). In middle age, asthma is more commonly adult-onset, sometimes referred to as “late-onset asthma,” with pathogenesis more inclined toward non-allergic inflammation and frequently accompanied by comorbidities such as obesity (7), hypertension (8), and metabolic syndrome (9–11).

The primary goals in managing chronic persistent asthma are to relieve symptoms, prevent acute exacerbations, and improve lung function and quality of life (12). Currently, inhaled corticosteroids are the most commonly used treatment (13). However, even with standardized therapy, some patients still fail to achieve satisfactory asthma control, and acute exacerbations continue to occur (14). As a traditional Chinese medicine therapy, acupuncture has demonstrated good efficacy and safety in various conditions, including obesity (15), migraine (16), anxiety (17), depression (18), and chronic gastrointestinal dysfunction (19). It has also been widely used to alleviate symptoms of chronic persistent asthma and to assist in improving airway inflammation and lung function (20).

Nevertheless, studies on the application of acupuncture in obese patients with asthma remain limited. It is still unclear whether acupuncture can simultaneously improve asthma symptoms and contribute to weight reduction. This study aims to address this gap and provide evidence for symptom control and weight management in obese patients with asthma.

## 2 Subjects and methods

### 2.1 Study population

*Study design*: Retrospective cohort study.

*Study population*: Asthma patients who received treatment at our hospital between January 2021 and December 2024.

Inclusion criteria:

1. Age ≥ 18 years
2. Diagnosis of chronic persistent asthma according to the *Global Initiative for Asthma (GINA) Guidelines for Asthma Management and Prevention (2022)* (21)
3. BMI ≥ 28 kg/m^2^ based on Chinese obesity criteria (22, 23)

Exclusion criteria:

1. History of acute asthma exacerbation or hospitalization within the past 4 weeks
2. Allergy or contraindications to acupuncture or related materials
3. Presence of severe systemic diseases, including advanced cancer or severe hepatic/renal insufficiency
4. Recent use of systemic corticosteroids or immunosuppressants
5. Pregnant or lactating women
6. Missing primary or secondary outcome data

Based on conventional statistical power assumptions (80% power, effect size d = 0.5), a *post hoc* assessment indicated that at least 64 participants per group were required for continuous variables and at least 63 participants per group for categorical variables. The present study included 225 participants in the control group and 190 participants in the treatment group, suggesting that the sample size was adequate for statistical analyses.

### 2.2 Treatment methods

*Control group*: Received conventional inhaled corticosteroid (ICS) therapy.

*Treatment group*: In addition to standard inhaled corticosteroid (ICS) therapy, patients in the treatment group received acupuncture, with each session lasting 30 min and administered three times per week. All patients received a standardized acupuncture protocol. Patients were placed in the lateral decubitus position. After routine disinfection, acupuncture was performed with perpendicular insertion at the back acupoints, with a needle depth of 1.0–1.5 cm. The acupoints included Danzhong (CV17), Dingchuan (EX-B1), Yunmen (LU2), Feishu (BL13), Dachangshu (BL25), Kongzui (LU6), Quchi (LI11), Hegu (LI4), Shangjuxu (ST37), and Xiajuxu (ST39) (20). Except for Danzhong, which was needled obliquely, all other acupoints were needled perpendicularly. During the procedure, the even reinforcing-reducing manipulation technique was applied, with two moxa cones per session. The needling sensation was required to achieve de qi (soreness, numbness, and distension), and needles were retained at each point for 30 min. All acupuncture treatments were administered by a licensed Traditional Chinese Medicine physician with more than 5 years of acupuncture experience and followed a standardized acupuncture protocol established by the department. No serious adverse events, including pneumothorax or other major complications, were observed during the treatment period.

Clinical and laboratory parameters were collected before treatment and at 6 months after treatment, including PEF (Peak Expiratory Flow), FEV1 (Forced Expiratory Volume in 1 s), FEV1/FVC (ratio of FEV1 to Forced Vital Capacity), ACT (Asthma Control Test), AQLQ (Asthma Quality of Life Questionnaire), IL-17 (Interleukin-17), CRP (C-reactive protein), and BMI (Body Mass Index).

It should be noted that no sham acupuncture control group was included in this study. Therefore, it cannot be determined whether the improvements in the collected indicators were solely attributable to acupuncture itself.

We defined treatment adherence as receiving ≥80% of scheduled acupuncture sessions. Patients meeting this criterion were included in the final analysis.

### 2.3 Outcome measures

*Primary outcome*: Based on the minimal clinically important difference (MCID) of the ACT (24), an improvement of ≥3 points was considered effective asthma control. An increase of <3 points was regarded as not achieving significant improvement in asthma control.

*Secondary outcome*: BMI change (post-treatment BMI - pre-treatment BMI).

### 2.4 Data analysis

All data were analyzed using R version 4.4. Continuous variables were first tested for normality. Data with a normal distribution were expressed as mean ± standard deviation, while non-normally distributed data were presented as median (interquartile range). Between-group comparisons were performed using the independent samples t-test or the Mann–Whitney U test. Categorical variables were expressed as frequency (percentage), and comparisons between groups were conducted using the chi-square test or Fisher’s exact test. Propensity score matching (PSM) was applied to control for baseline covariate imbalances between groups. Nearest-neighbor matching was performed at a 1:1 ratio between the treatment and control groups, with a caliper width of 0.1 of the standard deviation of the logit of the propensity score to reduce poor matches. Standardized mean differences (SMDs) were used to assess covariate balance. Multivariate logistic regression and multiple linear regression models were constructed using the primary and secondary outcomes as dependent variables, respectively. Model 1 included only the treatment method. Model 2 was adjusted for age, sex, and baseline BMI. Model 3 was further adjusted for age, sex, baseline BMI, male waist circumference, female waist circumference, smoking status, asthma severity, history of hospitalization, frequency, comorbidities, baseline PEF, baseline FEV1, baseline FEV1/FVC, baseline ACT, baseline AQLQ, baseline IL-17, and baseline CRP. A response model was developed to estimate asthma control outcomes, with the model formula calculated as a weighted total score based on the regression coefficients of significant factors. The discriminative ability of the model was evaluated using the receiver operating characteristic (ROC) curve, and the area under the curve (AUC), sensitivity, specificity, and optimal cutoff value were calculated. Bootstrap resampling was used for internal validation, with 1,000 iterations to calculate the mean AUC and standard deviation (SD). Additionally, stratified analyses were conducted to assess the predictive performance of the response model across different subgroups. Forest plots were used to visualize effect sizes (odds ratios, OR) and their 95% confidence intervals. The significance level was set at *α* = 0.05, with *p* < 0.05 considered statistically significant. When missing data were less than 15%, multiple imputation was applied; when missing data were ≥15%, the variable was excluded directly. Specifically, the number and proportion of missing data for each variable are presented in Supplementary Table 1. No missing data were observed for age and sex, and the overall proportion of missing data across other variables was low (0–9.88%).

## 3 Results

### 3.1 Baseline characteristics of the control and treatment groups

Among the 236 patients who initially received acupuncture treatment, 46 did not complete the predefined treatment or did not meet the adherence criteria. Ultimately, 190 patients (80.5%) achieved ≥80% treatment adherence and were included in the final analysis. A total of 415 patients were ultimately included in this study, with 225 in the control group and 190 in the treatment group. There were no significant differences between the two groups in terms of age, sex, disease duration, and other factors. Overall, the baseline characteristics were well balanced and comparable, providing a reliable foundation for subsequent analyses (Table 1). After PSM, both the control and treatment groups included 165 patients. Baseline characteristics were well balanced between the two groups, and all standardized mean differences (SMDs) of covariates after matching were less than 0.1, indicating adequate covariate balance (Supplementary Table 2; Supplementary Figure 1).

**Table 1:** Baseline characteristics of the control and treatment groups.

Characteristic	TOTAL (*n* = 415)	Control group (*n* = 225)	Treatment group (*n* = 190)	*p*-value
Age	49 (44–55)	49 (44–56)	49 (44–54)	0.617
Sex (Male)	185 (44.58%)	102 (45.33%)	83 (43.68%)	0.667
BMI	31.1 (28.8–32.9)	31.2 (29.0–33.0)	30.8 (28.6–32.8)	0.461
Waist circumference				
Male	94.9 (92.5–97.5)	94.9 (92.7–97.4)	94.9 (92.5–97.5)	0.91
Female	90.1 (87.1–94.1)	90.1 (87.3–94.1)	90.1 (87.1–93.8)	0.297
Smoking status				0.061
Never	297 (71.57%)	152 (67.56%)	145 (76.32%)	
Former	94 (22.65%)	61 (27.11%)	33 (17.37%)	
Current	24 (5.78%)	12 (5.33%)	12 (6.32%)	
Asthma severity classification				0.6065
Mild	154 (37.11%)	80 (35.56%)	74 (38.95%)	
Moderate	204 (49.16%)	111 (49.33%)	93 (48.95%)	
Severe	57 (13.73%)	34 (15.11%)	23 (12.11%)	
History of hospitalization (Yes)	103 (24.82%)	59 (26.22%)	44 (23.16%)	0.5445
Frequency of asthma exacerbations in the past year				0.9308
0	182 (43.86%)	98 (43.56%)	84 (44.21%)	
1–2	161 (38.8%)	89 (39.56%)	72 (37.89%)	
≥3	72 (17.35%)	38 (16.89%)	34 (17.89%)	
Comorbidities (Yes)	173 (41.69%)	93 (41.33%)	80 (42.11%)	0.953

### 3.2 Differences in clinical and laboratory indicators before and after treatment

The results showed that there were no significant differences in all indicators between the two groups before treatment. After treatment, the treatment group had significantly higher levels of PEF, FEV1, FEV1/FVC, ACT, and AQLQ compared to the control group. In contrast, inflammatory markers (IL-17 and CRP) were significantly lower in the treatment group. Additionally, the overall BMI after treatment was significantly lower in the treatment group than in the control group (Table 2). After treatment, the ACT score increased by 4 points in the control group and by 5 points in the treatment group, showing an overall improvement in both groups. The improvement was slightly greater in the treatment group, with a between-group difference of 1 point. BMI decreased by 0.7 kg/m^2^ in the control group and by 1.2 kg/m^2^ in the treatment group, indicating a greater reduction in the treatment group.

**Table 2:** Differences in clinical and laboratory parameters before and after treatment between the control and treatment groups.

PEF	TOTAL (*n* = 415)	Control group (*n* = 225)	Treatment group (*n* = 190)	*p*-value
Base	72.2 (69.0–75.5)	72.1 (68.7–76.1)	72.4 (69.2–75.1)	0.981
Post	78.3 (74.7–81.9)	77.5 (74.1–80.6)	79.3 (75.1–82.3)	0.00503
FEV1				
Base	69.0 (65.6–72.8)	68.6 (65.8–72.2)	69.3 (65.3–73.0)	0.473
Post	78.8 (74.8–82.6)	77.5 (74.5–81.2)	80.8 (75.4–83.7)	7.09E-05
FEV1/FVC				
Base	0.67 (0.62–0.73)	0.67 (0.62–0.72)	0.68 (0.63–0.73)	0.514
Post	0.73 (0.68–0.79)	0.72 (0.67–0.77)	0.75 (0.70–0.79)	6.57E-05
ACT				
Base	16 (15–18)	16 (15–18)	16 (15–18)	0.67
Post	20 (19–22)	20 (19–21)	21 (19–22)	0.0342
AQLQ				
Base	4.3 (3.9–4.7)	4.3 (3.9–4.7)	4.3 (3.8–4.7)	0.952
Post	5.1 (4.8–5.5)	5.0 (4.7–5.4)	5.2 (4.9–5.6)	0.000344
IL-17 (pg/mL)				
Base	29.0 (24.7–32.7)	29.0 (24.7–32.6)	29.4 (24.7–32.8)	0.873
Post	22.6 (19.5–26.3)	23.6 (19.9–27.0)	22.1 (19.2–25.4)	0.0384
CRP (mg/L)				
Base	5.5 (4.0–6.8)	5.6 (4.0–6.7)	5.4 (4.1–7.2)	0.734
Post	4.3 (2.8–5.7)	4.5 (2.9–5.9)	4.1 (2.7–5.4)	0.04
BMI change				
Base	30.7 (28.7–32.8)	30.8 (29.0–32.4)	30.7 (28.7–32.8)	0.677
Post	29.8 (28.4–31.3)	30.1 (28.7–31.5)	29.5 (28.2–31.1)	0.0326

PEF, Peak Expiratory Flow; FEV1, Forced Expiratory Volume in 1 Second; FVC, Forced Vital Capacity; FEV1/FVC, Ratio of FEV1 to FVC; ACT, Asthma Control Test; AQLQ, Asthma Quality of Life Questionnaire; IL-17, Interleukin-17; CRP, C-Reactive Protein; BMI, Body Mass Index.

### 3.3 Multivariate logistic regression analysis of the association between treatment method and asthma control

In the unadjusted Model 1, the treatment method showed a significant positive association with asthma control (OR = 1.141, *p* = 0.006, 95%CI: 1.039–1.254). After further adjustment for age, sex, and BMI in Model 2, this positive association remained significant (OR = 1.139, *p* = 0.007, 95%CI: 1.037–1.252). In Model 3, which included additional clinical and laboratory variables, the result remained stable (OR = 1.137, *p* = 0.008, 95%CI: 1.035–1.250). These findings suggest that the positive association between treatment method and asthma control is not influenced by age, sex, or baseline laboratory indicators (Table 3). We performed the same analysis on the PSM-matched dataset. The results showed that the positive association remained stable and statistically significant, suggesting that after controlling for selection bias, the association still held, demonstrating good robustness of the findings (Supplementary Table 3). To verify the robustness of these findings, we performed a complete-case analysis. The results remained consistent, showing a stable positive association between treatment method and asthma control, and no substantial changes were observed across different adjustment models (Supplementary Table 4).

**Table 3:** Multivariable logistic regression analysis of the independent effect of treatment on asthma control.

Term	Model 1				Model 2				Model 3			
	*p* value	OR	CI-lower	CI-upper	*p* value	OR	CI-lower	CI-upper	*p* value	OR	CI-lower	CI-upper
Method	0.006	1.141	1.039	1.254	0.007	1.139	1.037	1.252	0.008	1.137	1.035	1.250
Age	-	-	-	-	0.069	0.993	0.986	1.000	0.086	0.994	0.987	1.001
Sex	-	-	-	-	0.153	1.071	0.975	1.177	0.087	1.086	0.988	1.194
BMI	-	-	-	-	0.639	0.996	0.980	1.012	0.710	0.997	0.981	1.013
Waist circumference	-	-	-	-	-	-	-	-	0.480	1.016	0.973	1.060
Smoking status	-	-	-	-	-	-	-	-	0.825	0.991	0.914	1.074
Asthma severity	-	-	-	-	-	-	-	-	0.014	0.915	0.853	0.982
History of hospitalization	-	-	-	-	-	-	-	-	0.666	0.976	0.876	1.088
Frequency	-	-	-	-	-	-	-	-	0.924	1.003	0.941	1.069
Comorbidities	-	-	-	-	-	-	-	-	0.011	0.883	0.802	0.972
PEF	-	-	-	-	-	-	-	-	0.357	1.004	0.995	1.013
FEV1	-	-	-	-	-	-	-	-	0.020	1.013	1.002	1.025
FEV1/FVC	-	-	-	-	-	-	-	-	0.421	0.775	0.417	1.441
ACT	-	-	-	-	-	-	-	-	0.713	0.996	0.972	1.020
AQLQ	-	-	-	-	-	-	-	-	0.963	1.002	0.928	1.082
IL-17	-	-	-	-	-	-	-	-	0.651	0.998	0.990	1.006
CRP	-	-	-	-	-	-	-	-	0.026	0.968	0.940	0.996

PEF, Peak Expiratory Flow; FEV1, Forced Expiratory Volume in 1 Second; FVC, Forced Vital Capacity; FEV1/FVC, Ratio of FEV1 to FVC; ACT, Asthma Control Test; AQLQ, Asthma Quality of Life Questionnaire; IL-17, Interleukin-17; CRP, C-Reactive Protein; BMI – Body Mass Index.

### 3.4 Multiple linear regression analysis of the association between treatment method and BMI changes

In the unadjusted Model 1, the treatment method showed a significant negative association with BMI change (*β* = −0.125, *p* = 0.025). This negative association remained significant in both Model 2 and Model 3 (Model 2: *β* = −0.126, *p* = 0.024; Model 3: *β* = −0.141, *p* = 0.013). These results indicate that the treatment method is significantly associated with BMI reduction and is not affected by baseline variables (Table 4).

**Table 4:** Multivariable linear regression analysis of the independent effect of treatment on BMI change.

Term	Model 1		Model 2		Model 3	
	*β*	*p* value	*β*	*p* value	*β*	*p* value
Method	−0.125	0.025	−0.126	0.024	−0.141	0.013
Age	-	-	−0.003	0.548	−0.003	0.473
Sex	-	-	0.011	0.846	−0.151	0.278
BMI	-	-	−0.005	0.618	−0.004	0.647
Waist circumference	-	-	-	-	0.031	0.226
Smoking status	-	-	-	-	−0.023	0.636
Asthma severity	-	-	-	-	0.040	0.344
History of hospitalization	-	-	-	-	−0.018	0.787
Frequency	-	-	-	-	0.036	0.342
Comorbidities	-	-	-	-	0.043	0.459
PEF	-	-	-	-	−0.012	0.032
FEV1	-	-	-	-	−0.008	0.263
FEV1/FVC	-	-	-	-	0.101	0.788
ACT	-	-	-	-	0.007	0.647
AQLQ	-	-	-	-	−0.022	0.637
IL-17	-	-	-	-	0.008	0.108
CRP	-	-	-	-	−0.001	0.976

PEF, Peak Expiratory Flow; FEV1, Forced Expiratory Volume in 1 Second; FVC, Forced Vital Capacity; FEV1/FVC, Ratio of FEV1 to FVC; ACT, Asthma Control Test; AQLQ, Asthma Quality of Life Questionnaire; IL-17, Interleukin-17; CRP, C-Reactive Protein; BMI, Body Mass Index.

### 3.5 Construction of the response model

In Model 3, where asthma control was the dependent variable, in addition to the treatment method, asthma severity, comorbidities, baseline FEV1, and baseline CRP levels were also significantly associated with asthma control. To accurately predict asthma control outcomes, a response model was constructed using the coefficients from Model 3. The formula is as follows:

Response Model = Method × 0.129 + Asthma severity × (−0.089) + Comorbidities × (−0.124) + FEV1 × 0.013 + Baseline CRP × (−0.033).

**Figure 1:**
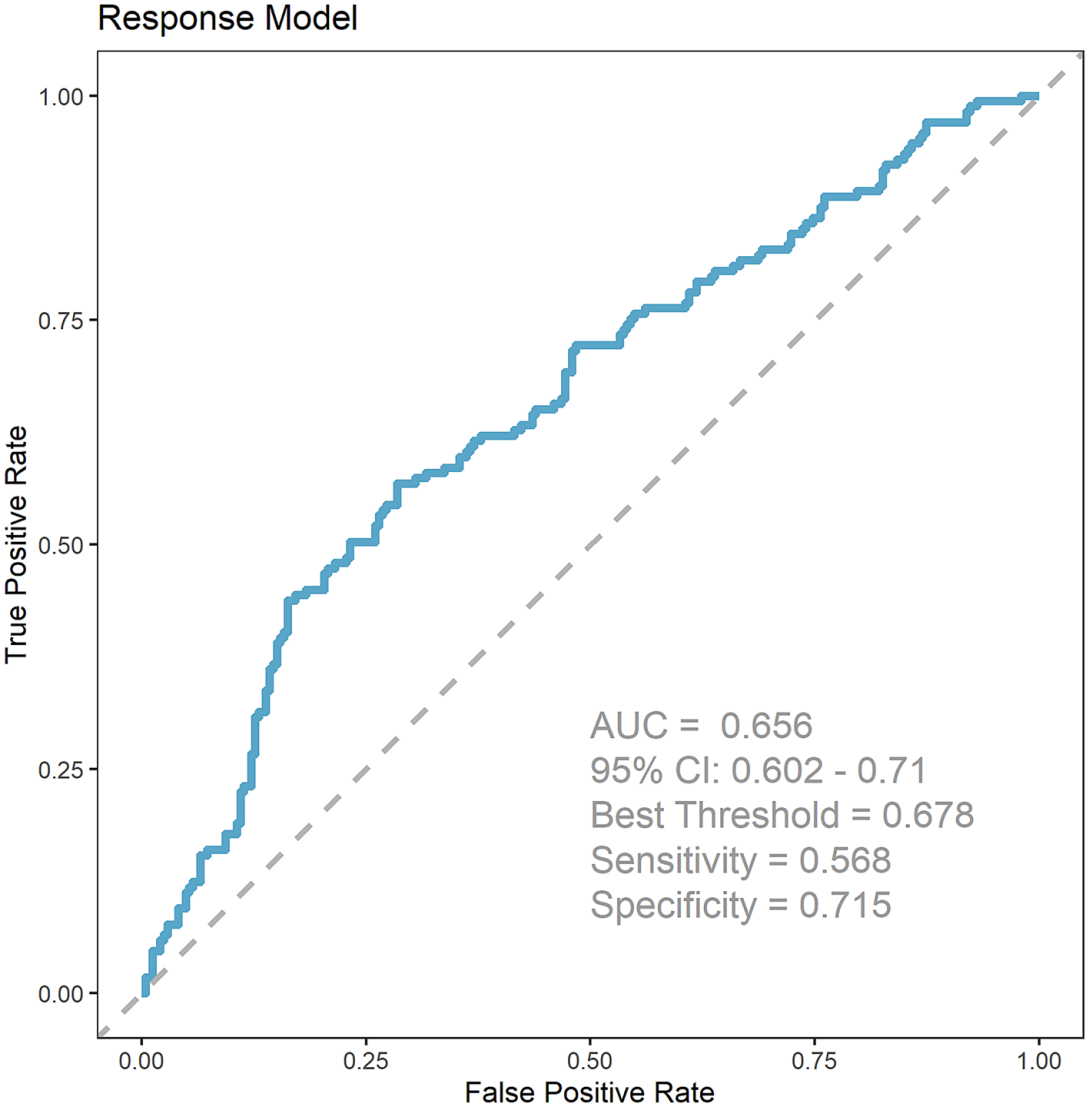
Receiver operating characteristic (ROC) curve of the response model.

The ROC curve showed that the AUC of the response model was 0.656, with a cutoff value of 0.678, sensitivity of 0.568, and specificity of 0.715. Bootstrap internal validation (1,000 resamples) yielded a mean AUC of 0.656 (SD = 0.027), and the optimism-corrected AUC was 0.655, indicating stable model performance with minimal overfitting. These results indicate that the model has moderate discriminative ability. When the patient’s response model score exceeds this threshold, there is a higher probability of achieving effective asthma control (Figure 1).

### 3.6 Stratified analysis

**Figure 2:**
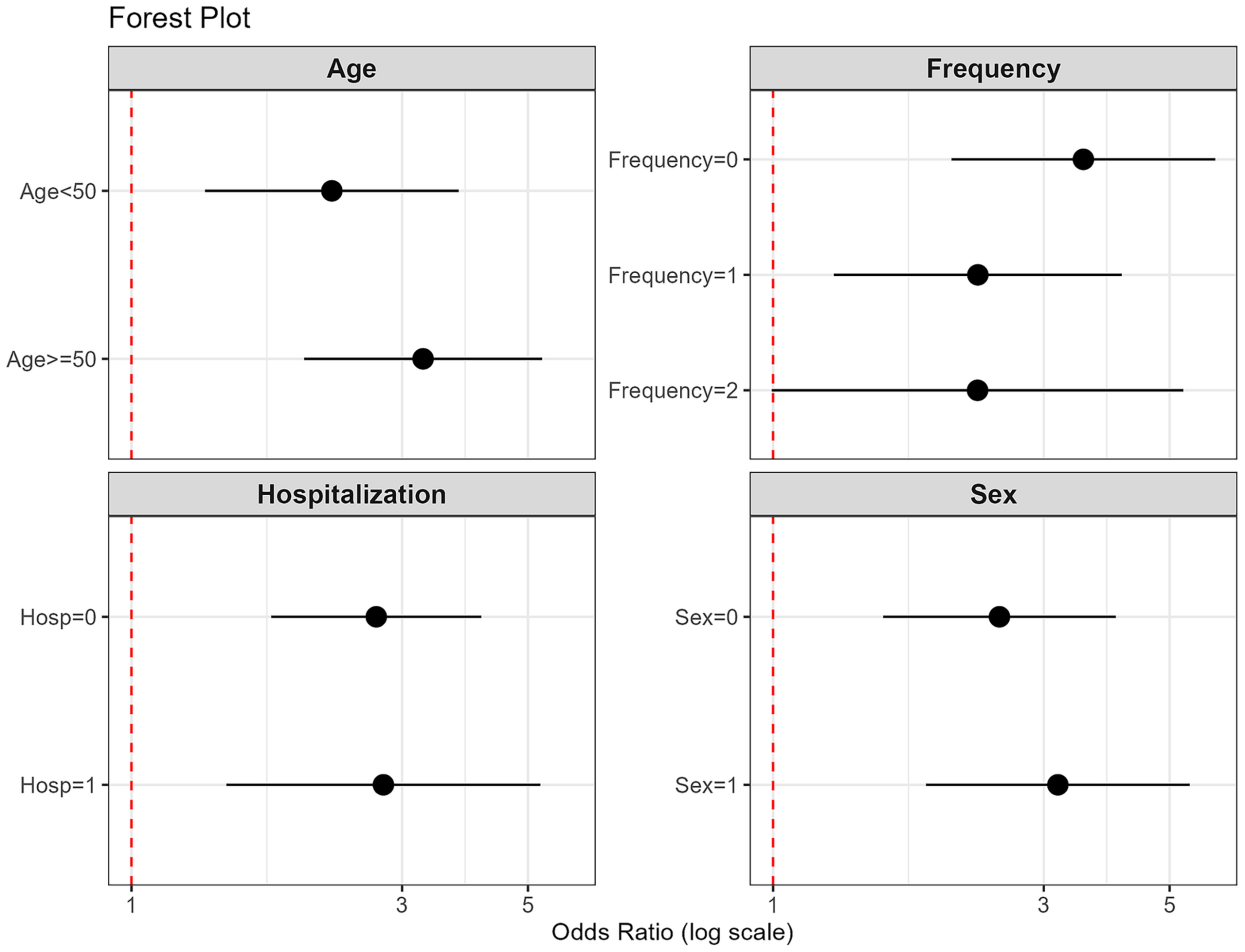
Forest plot of stratified analysis.

Further stratified analysis of the response model showed that its effect was stronger in patients aged ≥50 years (OR = 3.266, *p* < 0.001), while it was slightly lower in those aged <50 years (OR = 2.255, *p* = 0.002). The effect was stronger in males (OR = 3.177, *p* < 0.001) and slightly lower in females (OR = 2.506, *p* < 0.001). The strongest effect was observed in patients with no acute exacerbations in the past year (OR = 3.523, *p* < 0.001), followed by those with 1–2 exacerbations (OR = 2.296, *p* = 0.006). Notably, in patients with ≥3 exacerbations, the association did not reach statistical significance (OR = 2.293, *p* = 0.055). Regardless of prior hospitalization history, the model showed significant and comparable effect sizes (with hospitalization history: OR = 2.780, *p* = 0.002; without hospitalization history: OR = 2.701, *p* < 0.001) (Table 5; Figure 2).

**Table 5:** Stratified analysis.

Subgroup	*p* value	OR	CI-lower	CI-upper
Age ≥ 50	< 0.001	3.266	2.015	5.294
Age<50	0.002	2.255	1.348	3.774
Sex = Male	< 0.001	3.177	1.860	5.427
Sex = Female	< 0.001	2.506	1.563	4.018
Frequency = 0	< 0.001	3.523	2.063	6.017
Frequency = 1–2	0.006	2.296	1.280	4.119
Frequency = 3	0.055	2.293	0.995	5.286
History of hospitalization = Yes	0.002	2.780	1.470	5.258
History of hospitalization = No	< 0.001	2.701	1.763	4.138

## 4 Discussion

Our study found that acupuncture is significantly associated with effective asthma control in obese patients with asthma. Current research on the mechanisms of acupuncture in asthma mainly focuses on two pathways: neural regulation and molecular immune modulation. Acupuncture plays an important role in neurotransmitter signaling. Fei-Xuan et al. demonstrated that acupuncture activates vagal nerve fibers, promoting the release of acetylcholine from nerve terminals. This stimulation binds to α7 nicotinic acetylcholine receptors (α7nAChR) on immune cells, subsequently inhibiting the release of pro-inflammatory factors via intracellular signaling pathways (25). The molecular immune modulation pathway can be summarized as the regulation of inflammatory cytokines to reduce airway inflammation and restore immune balance, thereby improving asthma. Ying et al. experimentally demonstrated that acupuncture reduced Th17 cell cytokines, including IL-17A, IL-17F, and IL-22, in the serum of mice, suppressing airway hyperresponsiveness, pulmonary inflammation, and mucus secretion in experimental asthma models (26). Vagal nerve stimulation is also closely related to metabolic processes such as appetite and energy expenditure. Tianxiao et al. confirmed that vagal nerve stimulation significantly reduced body weight in rats and piglets, suppressed their appetite, and increased energy expenditure (27). Therefore, the vagus nerve–acetylcholine pathway not only participates in anti-inflammatory responses but may also play a key role in metabolic regulation and weight control. Similarly, inflammatory pathways are highly associated with obesity. Acupuncture can reduce inflammation, alleviate obesity-related chronic low-grade inflammation, improve metabolic function, and to some extent promote weight loss. Fernando et al. demonstrated that the expression of IL-17A in visceral adipose tissue was significantly higher in women with morbid obesity compared to normal women (28). Thus, the Th17-related cytokine pathways regulated by acupuncture may represent a common mechanism for both asthma control and weight reduction.

The response model constructed in this study has important clinical significance. It integrates significant factors—including treatment method, asthma severity, comorbidities, baseline FEV1, and baseline CRP—into a single mathematical formula, providing a quantifiable and reproducible measure to evaluate treatment efficacy. Asthma control is influenced not only by treatment methods but also by numerous individual factors. Therefore, one cannot conclude that a patient will achieve effective asthma control solely by receiving acupuncture. In this context, the response model can be used to predict whether a patient is likely to achieve effective asthma control. By inputting baseline information for each patient, a response model score can be obtained. If the score is below the threshold, clinicians are advised to adjust the treatment plan or enhance follow-up. The response model demonstrated a modest AUC value but relatively higher specificity (0.715), indicating a better ability to identify patients with poor asthma control. On this basis, the model may serve as an exploratory tool at the research level to assist in identifying potentially high-risk individuals and provide complementary information to traditional clinical indicators.

Stratified analysis indicated that the response model had stronger effects in patients aged ≥50 years, males, and those without acute exacerbations in the past year. This may be because older patients tend to have more comorbidities, and since comorbidities are included as a variable in our response model, the model’s explanatory power is enhanced. In patients with relatively stable disease, neuro-immune regulation is more controllable and inflammation levels are more stable, making the effects of acupuncture easier for the model to capture. This also suggests that in complex or severe cases, a more comprehensive assessment incorporating additional clinical indicators is still necessary.

The between-group differences in ACT and BMI after treatment were relatively small, suggesting a modest magnitude of improvement associated with acupuncture. From a real-world clinical perspective, these changes may be insufficient to produce meaningful shifts in symptom classification or substantial benefits in body weight–related outcomes. Therefore, their clinical relevance may be limited, and the findings are better interpreted as a trend rather than a clinically substantial therapeutic effect.

This study also has certain limitations. As a single-center retrospective study, the representativeness of the sample is limited, and there may be some degree of selection bias. Important lifestyle factors that may influence asthma control outcomes, such as diet and physical activity, were not fully collected or adjusted for in this study. The response model demonstrated a sensitivity of 0.568 and specificity of 0.715, indicating relatively good ability to exclude non-responders; therefore, it is more suitable as an auxiliary tool for clinical risk stratification. It should be noted that although only patients who met the predefined adherence criterion (≥80%) were included, there was still some variability in actual treatment exposure among these patients, which may constitute a potential source of variation. Although the acupuncture plus ICS group showed better clinical outcomes than the ICS-only group, these improvements may also be attributed to other external factors, such as differences in nursing care and patient-related characteristics. Future studies incorporating sham acupuncture as a control are needed to further clarify the independent contribution of acupuncture to treatment outcomes.

## 5 Conclusion

Acupuncture is closely associated with improvements in pulmonary function, asthma-related patient-reported outcomes, inflammatory markers, and BMI in obese patients with asthma. After accounting for baseline confounding factors, acupuncture remains significantly associated with effective asthma control and BMI changes. The response model shows a certain level of discriminative ability and may assist in predicting individual patients’ treatment response. However, due to its modest AUC and limited predictive performance, it should be considered an exploratory tool rather than a clinical decision-making tool. In subgroup analyses, relatively stronger associations were observed in patients aged ≥50 years, males, and those without acute exacerbations in the past year. However, due to the absence of a sham acupuncture control group in this study, the observed benefits cannot be fully attributed to the specific effects of acupuncture itself, and further studies are needed to validate these findings. This study provides a basis for individualized treatment and precise risk stratification.

## Data Availability

The raw data supporting the conclusions of this article will be made available by the authors, without undue reservation.

## Supplementary material

SUPPLEMENTARY FIGURE 1 Standardized mean differences of covariates before and after propensity score matching.
