# Supplementary figures and images for: Effect of acupuncture on asthma control and body weight changes in obese patients with asthma
Source: Front Med (Lausanne). 2026 Jul 3;13:1861336. doi: 10.3389/fmed.2026.1861336 (PMC13376247; doi:10.3389/fmed.2026.1861336)

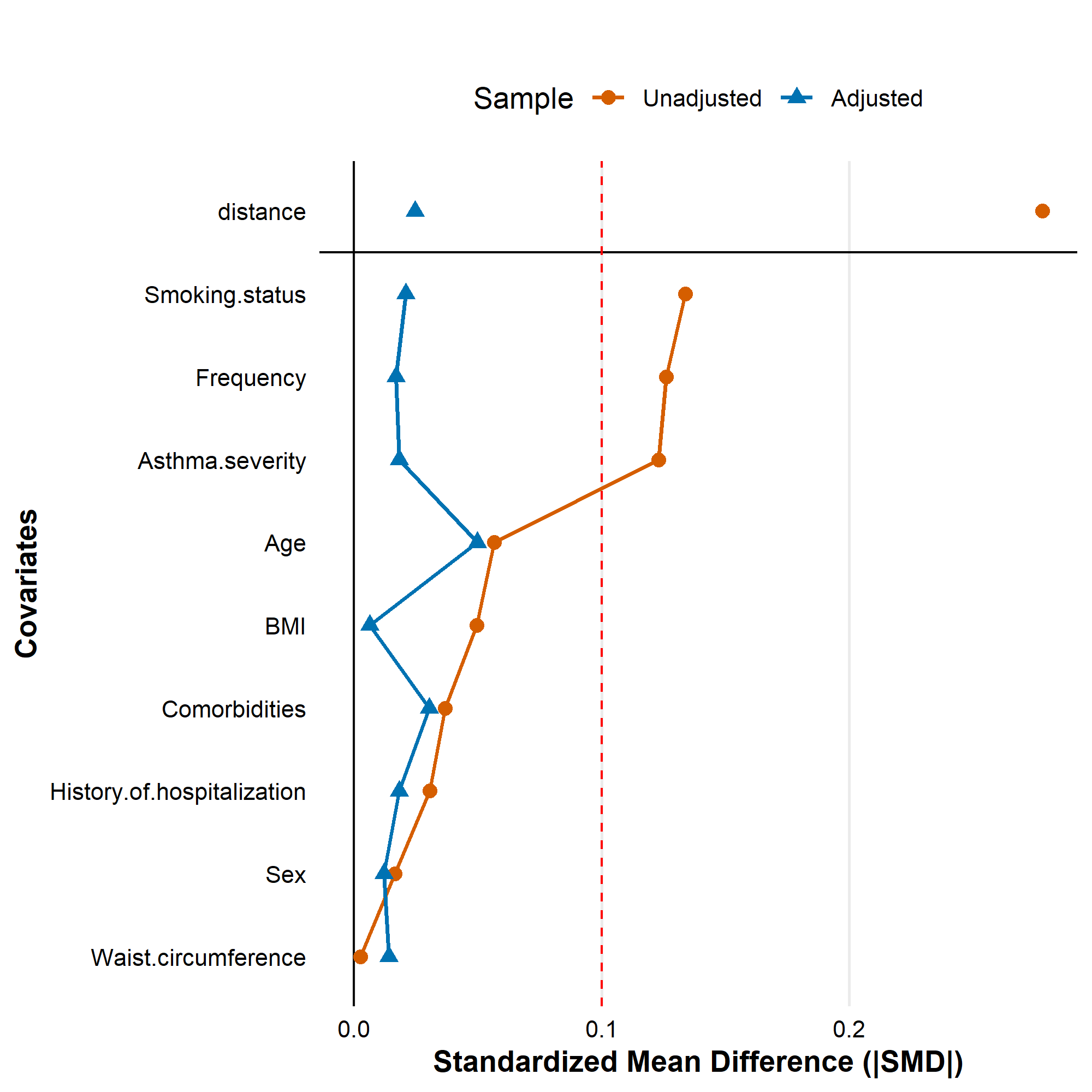

Supplement: SUPPLEMENTARY FIGURE 1 — Standardized mean differences of covariates before and after propensity score matching. [file Supplementary_Figure_1.tif]
