# Supplementary material for: Effect of acupuncture on asthma control and body weight changes in obese patients with asthma
Source: Front Med (Lausanne). 2026 Jul 3;13:1861336. doi: 10.3389/fmed.2026.1861336 (PMC13376247; doi:10.3389/fmed.2026.1861336)
Supplement: Supplementary file 2 [file Supplementary_file_1.docx]

| **Supplementary Table. 1 Number and proportion of missing cases for each variable** | | |
| --- | --- | --- |
| **Variables** | **n** | **Percent (%)** |
| **Age** | 0 | 0.00 |
| **Sex** | 0 | 0.00 |
| **BMI** | 6 | 1.45 |
| **Smoking.status** | 26 | 6.27 |
| **Asthma.severity** | 41 | 9.88 |
| **History.of.hospitalization** | 27 | 6.51 |
| **Frequency** | 10 | 2.41 |
| **Comorbidities** | 8 | 1.93 |
| **PEF** | 26 | 6.27 |
| **FEV1** | 28 | 6.75 |
| **FEV1.FVC** | 21 | 5.06 |
| **ACT** | 2 | 0.48 |
| **AQLQ** | 16 | 3.86 |
| **CRP** | 32 | 7.71 |
| **IL.17** | 34 | 8.19 |
| **Waist.circumference** | 37 | 8.92 |

| **Supplementary Table. 2 Baseline characteristics of the control and treatment groups after Propensity Score Matching (PSM)** | | | | |
| --- | --- | --- | --- | --- |
|  | **TOTAL (n=330)** | **Control group (n=165)** | **Treatment group (n=165)** | **P-value** |
| **Age** | 50 (44-55) | 50 (43-55) | 49 (44-54) | 0.673 |
| **Sex (Male)** | 148 (44.8%) | 73 (44.2%) | 75 (45.5%) | 0.912 |
| **BMI** | 31.2 (28.9-32.9) | 31.2 (29-33.2) | 30.9 (28.7-32.8) | 0.8 |
| **Waist circumference** |  |  |  |  |
| **Male** | 94.9 (94.3-95.5) | 94.9 (94.2-95.5) | 94.9 (94.4-95.5) | 0.843 |
| **Female** | 90.1 (89.2-90.9) | 90 (89-91) | 90.1 (89.4-90.8) | 0.481 |
| **Smoking status** |  |  |  | 0.262 |
| Never | 244 (73.9%) | 119 (72.1%) | 125 (75.8%) |  |
| Former | 72 (21.8%) | 41 (24.8%) | 31 (18.8%) |  |
| Current | 14 (4.2%) | 5 (3%) | 9 (5.5%) |  |
| **Asthma severity classification** |  |  |  | 0.619 |
| Mild | 115 (34.8%) | 59 (35.8%) | 56 (33.9%) |  |
| Moderate | 164 (49.7%) | 78 (47.3%) | 86 (52.1%) |  |
| Severe | 51 (15.5%) | 28 (17%) | 23 (13.9%) |  |
| **History of hospitalization (Yes)** | 75 (22.7%) | 36 (21.8%) | 39 (23.6%) | 0.793 |
| **Frequency of asthma exacerbations in the past year** |  |  |  | 0.117 |
| 0 | 140 (42.4%) | 75 (45.5%) | 65 (39.4%) |  |
| 1_2 | 126 (38.2%) | 54 (32.7%) | 72 (43.6%) |  |
| >=3 | 64 (19.4%) | 36 (21.8%) | 28 (17%) |  |
| **Comorbidities (Yes)** | 141 (42.7%) | 68 (41.2%) | 73 (44.2%) | 0.656 |

| **Supplementary Table. 3 Multivariable logistic regression analysis of the independent effect of treatment on asthma control after Propensity Score Matching (PSM)** | | | | | | | | | | | | |
| --- | --- | --- | --- | --- | --- | --- | --- | --- | --- | --- | --- | --- |
|  | **Model 1** | | | | **Model 2** | | | | **Model 3** | | | |
| **Term** | **P value** | **OR** | **CI-lower** | **CI-upper** | **P value** | **OR** | **CI-lower** | **CI-upper** | **P value** | **OR** | **CI-lower** | **CI-upper** |
| **Method** | 0.005 | 1.164 | 1.047 | 1.293 | 0.006 | 1.160 | 1.044 | 1.289 | 0.008 | 1.151 | 1.038 | 1.277 |
| **Age** | - | - | - | - | 0.162 | 0.994 | 0.986 | 1.002 | 0.163 | 0.994 | 0.986 | 1.002 |
| **Sex** | - | - | - | - | 0.129 | 1.086 | 0.977 | 1.208 | 0.606 | 1.072 | 0.823 | 1.397 |
| **BMI** | - | - | - | - | 0.799 | 0.998 | 0.980 | 1.016 | 0.966 | 1.000 | 0.982 | 1.018 |
| **Waist circumference** | - | - | - | - | - | - | - | - | 0.816 | 1.006 | 0.958 | 1.056 |
| **Smoking status** | - | - | - | - | - | - | - | - | 0.555 | 0.971 | 0.882 | 1.070 |
| **Asthma severity** | - | - | - | - | - | - | - | - | 0.013 | 0.906 | 0.839 | 0.979 |
| **History of hospitalization** | - | - | - | - | - | - | - | - | 0.675 | 0.974 | 0.859 | 1.103 |
| **Frequency** | - | - | - | - | - | - | - | - | 0.961 | 1.002 | 0.934 | 1.074 |
| **Comorbidities** | - | - | - | - | - | - | - | - | 0.002 | 0.846 | 0.760 | 0.942 |
| **PEF** | - | - | - | - | - | - | - | - | 0.849 | 1.001 | 0.991 | 1.011 |
| **FEV1** | - | - | - | - | - | - | - | - | 0.024 | 1.014 | 1.002 | 1.027 |
| **FEV1/FVC** | - | - | - | - | - | - | - | - | 0.197 | 0.640 | 0.325 | 1.259 |
| **ACT** | - | - | - | - | - | - | - | - | 0.589 | 0.993 | 0.968 | 1.019 |
| **AQLQ** | - | - | - | - | - | - | - | - | 0.554 | 0.975 | 0.898 | 1.060 |
| **IL-17** | - | - | - | - | - | - | - | - | 0.722 | 1.002 | 0.993 | 1.010 |
| **CRP** | - | - | - | - | - | - | - | - | 0.028 | 0.964 | 0.934 | 0.996 |

| **Supplementary Table. 4 Multivariable logistic regression analysis of the independent effect of treatment on asthma control in the complete-case dataset** | | | | | | | | | | | | |
| --- | --- | --- | --- | --- | --- | --- | --- | --- | --- | --- | --- | --- |
|  | **Model 1** | | | | **Model 2** | | | | **Model 3** | | | |
| **Term** | **P value** | **OR** | **CI-lower** | **CI-upper** | **P value** | **OR** | **CI-lower** | **CI-upper** | **P value** | **OR** | **CI-lower** | **CI-upper** |
| **Method** | 0.006 | 1.141 | 1.039 | 1.254 | 0.004 | 1.148 | 1.045 | 1.262 | 0.010 | 1.206 | 1.047 | 1.390 |
| **Age** | - | - | - | - | 0.045 | 0.993 | 0.986 | 1.000 | 0.201 | 0.993 | 0.983 | 1.004 |
| **Sex** | - | - | - | - | 0.177 | 1.068 | 0.971 | 1.174 | 0.285 | 0.826 | 0.583 | 1.171 |
| **BMI** | - | - | - | - | 0.537 | 0.995 | 0.979 | 1.011 | 0.249 | 0.986 | 0.963 | 1.010 |
| **Waist circumference** | - | - | - | - | - | - | - | - | 0.145 | 1.048 | 0.984 | 1.117 |
| **Smoking status** | - | - | - | - | - | - | - | - | 0.424 | 1.051 | 0.931 | 1.186 |
| **Asthma severity** | - | - | - | - | - | - | - | - | 0.039 | 0.896 | 0.807 | 0.994 |
| **History of hospitalization** | - | - | - | - | - | - | - | - | 0.736 | 0.974 | 0.836 | 1.134 |
| **Frequency** | - | - | - | - | - | - | - | - | 0.576 | 1.028 | 0.934 | 1.130 |
| **Comorbidities** | - | - | - | - | - | - | - | - | 0.242 | 0.918 | 0.797 | 1.059 |
| **PEF** | - | - | - | - | - | - | - | - | 0.799 | 1.002 | 0.988 | 1.016 |
| **FEV1** | - | - | - | - | - | - | - | - | 0.003 | 1.025 | 1.008 | 1.042 |
| **FEV1/FVC** | - | - | - | - | - | - | - | - | 0.547 | 1.299 | 0.555 | 3.041 |
| **ACT** | - | - | - | - | - | - | - | - | 0.490 | 0.988 | 0.955 | 1.022 |
| **AQLQ** | - | - | - | - | - | - | - | - | 0.642 | 0.972 | 0.863 | 1.095 |
| **IL-17** | - | - | - | - | - | - | - | - | 0.526 | 1.004 | 0.992 | 1.016 |
| **CRP** | - | - | - | - | - | - | - | - | 0.034 | 0.954 | 0.914 | 0.996 |
